# Analysis of Selected Phenolic Compounds in Organic, Pesticide-Free, Conventional Rice (*Oryza sativa* L.) Using LC-ESI-MS/MS

**DOI:** 10.3390/molecules24010067

**Published:** 2018-12-25

**Authors:** Mayakrishnan Prabakaran, Ill-Min Chung, Na-Young Son, Hee-Youn Chi, So-Yeon Kim, Yu-Jin Yang, Chang Kwon, Yeon-Ju An, Ateeque Ahmad, Seung-Hyun Kim

**Affiliations:** 1 Department of Crop Science, College of Sanghuh Life Science, Konkuk University, Seoul 05029, Korea; prabakarannitt@gmail.com (M.P.); imcim@konkuk.ac.kr (I.-M.C.); sonkittyy@konkuk.ac.kr (N.-Y.S.); chi1143@konkuk.ac.kr (H.-Y.C.); hellosy1@konkuk.ac.kr (S.-Y.K.); jin9031@konkuk.ac.kr (Y.-J.Y.); chang794@konkuk.ac.kr (C.K.); ayj3043@konkuk.ac.kr (Y.-J.A.); 2 Process Chemistry and Technology Department, CSIR-Central Institute of Medicinal and Aromatic Plants, Lucknow 226015, India; ateeque97@gmail.com

**Keywords:** rice (*Oryza sativa* L.), pesticide-free rice, conventional rice, organic rice, phenolic compound, LC-ESI-MS/MS

## Abstract

Rice (*Oryza sativa* L.) contains generous amounts of carbohydrates, proteins, vitamins, and dietary fibers, in addition to secondary metabolites such as phenols and flavonoids that act as antioxidants. The phenolic compounds detected in rice (organic rice (OR), conventional rice (CR), and pesticide-free rice (PFR)), namely, protocatechuic, gentisic, *p*-hydroxybenzoic, *p*-coumaric, ferulic, salicylic, and caffeic acids, are notable free radical scavengers. The sum of these phenolic compounds was found to be higher in PFR, followed by CR and OR (*p* < 0.0001), when the rice types were classified based on the farming system employed. In addition, significant differences were observed in the *p*-hydroxybenzoic acid levels for the OR and CR groups compared with the PFR groups (*p* < 0.01). Furthermore, greater quantities of *p*-coumaric acid were found in CR-08 and OR-02, although these groups contained overall higher and lower sums of phenolic compounds, respectively. Moreover, significance was observed in the sum of the phenolic compounds, although only small quantities were found in polished rice. Further research is thus required to provide a clearer picture regarding the phenolic profiles of different rice brands.

## 1. Introduction

*Oryza sativa* L., otherwise known as rice, is one of the most widely consumed staple foods, providing 20% of food calories worldwide. In particular, it is staple food in Asia and Latin America, where an average of 700 calories/day/person are consumed in the form of rice alone. Rice is rich in proteins and easily digestible carbohydrates, in addition to β-glucans; fats; fibers; and macrominerals such as phosphorus, potassium, calcium, and magnesium [1,2].

In addition, secondary metabolites are produced by plants from primary metabolites, and these compounds are non-essential for the growth and development of an organism [3]. Such secondary metabolites are a broad group of chemical components with various ecological functions, and examples include vanillic acid (found in *Paratecoma koraiensis),* hydroxybenzoic acid *(Citrus paradisi*), ferulic acid (cereal brans), and *p*-coumaric acid (*Triticum aestivum* L. and *Zea mays*). These compounds tend to be spread over the root, stem, bark, and leaves [4].

In this context, various studies carried out in pigmented and unpigmented rice have reported the presence of phenolic secondary metabolites, such as 3-hydroxybenzaldehyde, as well as gallic, protocatechic, *p*-hydrobenzoic, vanillic, caffeic, syringic, *p*-coumaric, and ferulic acids. In addition, the red, dark purple, and black pigmented rice species are rich in phenolics, tannin, flavones, and amino acids, with anthocyanins (i.e., cyanidin, malvidin, and pelagonidin) being the major pigments [5,6].

It has also been reported that different levels of pigments and phenolic compounds are produced by wild and organic pigmented rice varieties that are native to countries like Korea, Myanmar, Cambodia, and Thailand. In addition, studies have shown variations in the quantities of phenolic and other phytochemical constituents in rice varieties produced using different cropping systems, thereby indicating the importance of agricultural practice on plant metabolites [7,8].

In terms of their biological activity, phenolic compounds exhibiting a redox nature are known to act as free radical scavengers because of their hydrogen donor properties. This activity can be important in preventing various illnesses including cancer; diabetes; neuronal degeneration; and cardiovascular, pulmonary, and autoimmune diseases [9,10,11,12].

Such phenolic compounds are commonly synthesized through the shikimate, phenylpropanoid, and pentose phosphate pathways. For example, phenylalanine (PAL) produced via the shikimate pathway is incorporated into the phenylpropanoid pathway to synthesize various other phenolic compounds (Figure S1, see online supplementary material) [13,14]. During the phenylpropanoid pathway, PAL is subjected to a series of reactions (e.g., hydroxylation and methylation) to give an intermediate phenylpropanoid carbon skeleton [15], which ultimately leads to the formation of *p*-coumaric, caffeic, and ferulic acids, in addition to simple coumarins [16].

More specifically, the phenylpropanoid pathway produces *p*-coumarate from cinnamate, which is later involved in the synthesis of caffeic and chlorogenic acids, in addition to *ρ*-coumaroyl and malonyl CoA, genistein, flavonols, and anthocyanins [17]. Indeed, secondary metabolites such as phenylpropanoid, hydroxycinnamic acid, and stilbene protect the plants from various illnesses and stresses. Furthermore, salicylic acid is synthesized through the decarboxylation of cinnamic acid. It is closely related to plant disease and is associated with the synthesis of phenylpropanoids [18].

In recent decades, the general population has become increasingly concerned with their quality of life, which ultimately leads to improvements in health and the environment. More specifically, the consumption of residue-containing food products by infants and children is now deemed undesirable. Thus, to eradicate the use of organic synthetic fertilizers and pesticides, organic agriculture is becoming common in Korea. The use of organic manure has thus become more common to provide nutrition to plants [19,20]. Indeed, in the cases of eco-friendly rice production in Korea, the organic rice is produced without the use of synthetic pesticides and chemical fertilizers, and the pesticide-free rice is also produced by using up to a third of the recommended level of chemical fertilizers, as well as no pesticides. In a prior systematic review [21], strict organic dairy product consumption resulted in significantly reduced instances of eczema in infants; however, the difference in nutrition-related health outcomes resulting from consumption of organic or conventional foods still remains unclear.

Although various agricultural systems have been practiced to date, few comparative studies have been carried out to examine differences in the antioxidant phenolic compounds present in organic rice (OR), pesticide-free rice (PFR), and conventional rice (CR). Thus, we herein report our analysis of selected phenolic compounds in these three rice groups using liquid chromatography-electrospray ionization-tandem mass spectrometry (LC-ESI-MS/MS).

## 2. Results and Discussion

### 2.1. Comparison of the Phenolic Components Present in OR, PFR, and FR

**Figure 1:**
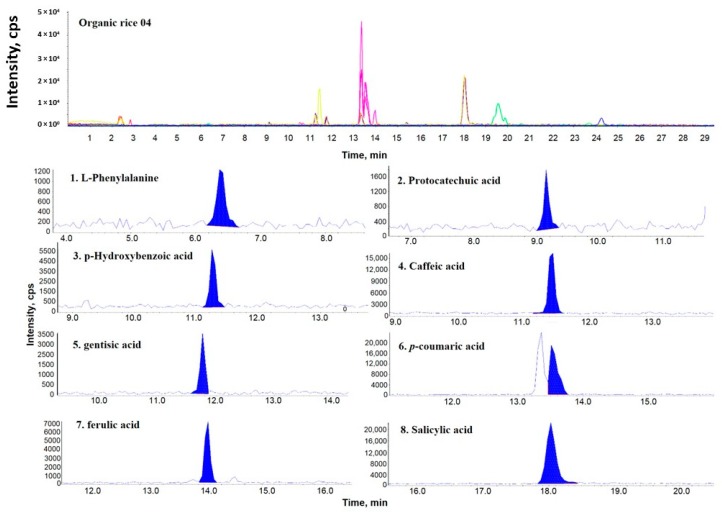
Representative multiple reaction monitoring (MRM) ion chromatogram of organic rice (OR)-04 and the corresponding extracted ion chromatograms of the detected phenolic compounds.

**Figure 2:**
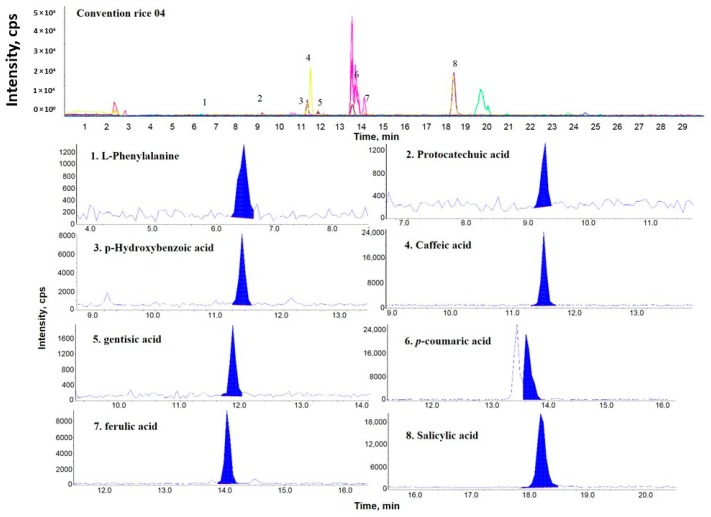
Representative MRM ion chromatogram of conventional rice (CR)-04 and the corresponding extracted ion chromatograms of the detected phenolic compounds.

**Figure 3:**
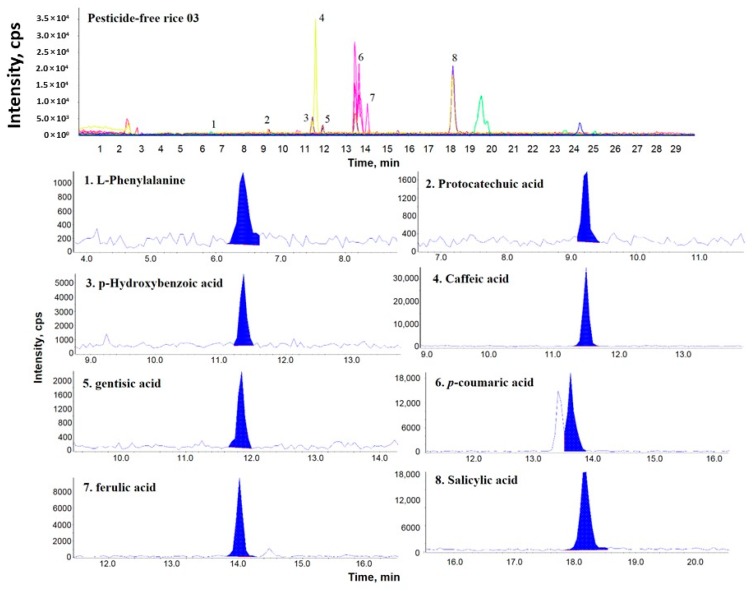
Representative MRM ion chromatogram of pesticide-free rice (PFR)-03 and the corresponding extracted ion chromatograms of the detected phenolic compounds.

Figure 1, Figure 2 and Figure 3 show representative multiple reaction monitoring (MRM) ion chromatograms for OR-04, CR-04, and PFR-03, in addition to ion chromatograms of the detected phenolic compounds.

The quantities of the various phenolic compounds detected when the different farming systems were employed are outlined in Table 1. As indicated, seven phenolic compounds were detected in OR, CR, and PFR, including protocatechuic, gentisic, *p*-hydroxybenzoic, *p*-coumaric, ferulic, salicylic, and caffeic acids. The sums of these phenolic compounds ranged from 1.20 μg/g (OR-02) to 3.73 μg/g (CR-08). This gave an average of 2.51 μg/g with a standard deviation of 0.65 μg/g.

**Figure 4:**
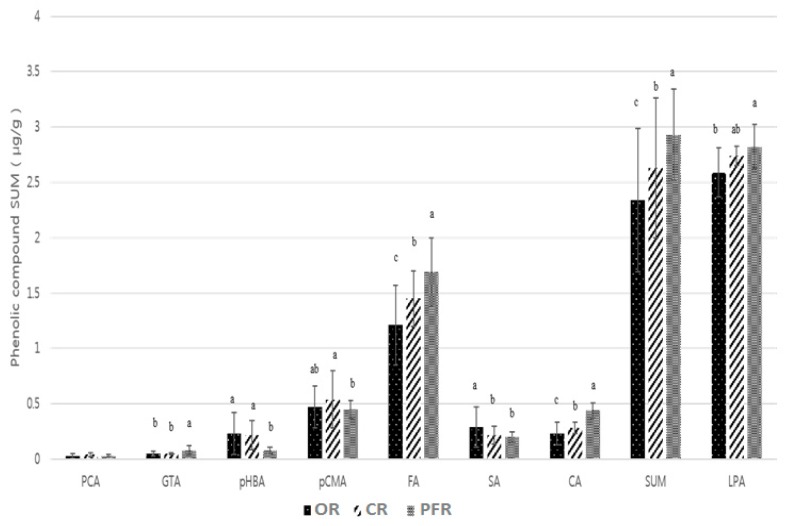
Distribution of phenolic compounds in OR, CR, and PFR. Letters a–c indicate a significant difference among the three groups (*p* < 0.05). Abbreviations: PCA (protocatechuic acid), GTA (gentisic acid), pHBA (p-hydroxybenzoic acid), pCMA (p-coumaric acid), FA (ferulic acid), SA (salicylic acid), CA (caffeic acid), LPA (l-phenylalanine), and ND (not detected).

Figure 4 shows the significant differences for the various phenolic compounds and farming systems. When classified according to the farming system, the highest sum of the phenolic compounds was found in PFR, followed by CR and OR (Figure 4) (*p* < 0.0001).

As indicated, in all farming system groups, phenylpropanoids such as ferulic, *p*-coumaric, and caffeic acids were detected, in addition to phenolic acids such as salicylic acid. The most abundant compound was ferulic acid, which supports the results of previous studies. In general, the highest contents of ferulic acid were found in polished and brown rice, with the value for brown rice being triple that found in polished rice. Similarly, ferulic acid was the most abundant phenolic compound in rice bran, with an absolute value seven times greater than that of polished rice [22]. More specifically, the ferulic acid contents of the various rice products decreased in the following order: bran > husk > brown rice > polished rice [23]. In addition, the phenolic content in polished rice was higher in the insoluble fraction (i.e., in the residue after methanol extraction) than in the soluble fraction (i.e., in the crude methanol extract), with ferulic acid being the most abundant component in both fractions [9].

Upon classification according to the farming system, the ferulic acid content decreased in the following order: PFR > CR > OR (*p* < 0.0001). In the case of *p*-coumaric acid, significant differences were observed for CR and PFR, while for caffeic acid, the content of this phenolic followed the same trend as for ferulic acid (*p* < 0.0001). In the case of salicylic acid, the highest significant difference was observed for OR (*p* < 0.001). Other phenolic compounds detected in the rice samples included p-hydroxybenzoic, gentisic, and protocatechuic acids. In the case of *p*-hydroxybenzoic acid, OR and CR showed higher significant differences compared with PFR (*p* < 0.01). However, in all groups, samples existed that were below the limit of detection (LOD). In addition, the mean values for the OR and CR groups were 0.23 and 0.22 μg/g, respectively, indicating no significant difference. This was partly the result of each group having at least one sample with a particularly high value (i.e., OR-07, OR-11, and CR-08). In terms of the gentisic acid content, this decreased in the following order: PFR > CR > OR (*p* < 0.0001). Again, both the OR and CR groups contained samples that were below the LOD. In the case of protocatechuic acid, no significant differences were observed between the groups (*p* > 0.05), although the values for more than half of the OR and CR groups were below the LOD.

The amino acid l-phenylalanine is a precursor of phenolic acid, producing cinnamic, *p*-coumaric, caffeic, and ferulic acids through the action of the phenylalanine ammonia lyase (PAL) enzyme [16,24,25,26]. l-Phenylalanine and l-tyrosine, which differ in the number of −OH groups, are produced by the action of the arogenate dehydrogenase (ADH) and arogenate dehydratase (ADT) enzymes within plastids. The cinnamic acid produced from l-phenylalanine by the action of PAL is converted to *p*-coumaric acid by CA4H, which is also produced from l-tyrosine by TAL (tyrosine ammonia lyase) [16,27,28,29,30]. However, in this study, only l-phenylalanine was detected, although this may be the result of the l-tyrosine quantities being below the LOD (LOD and LOQ (limits of quantification) for l-phenylalanine = 0.5 and 1 μg/mL, respectively). Differences in the required extraction conditions for these compounds may also influence their abundance in the analytical samples. Indeed, l-phenylalanine was only detected in one sample per OR and CR group, although it was present in all PFR samples. 

### 2.2. Comparison of the Phenolic Contents by Rice Samples

**Figure 5:**
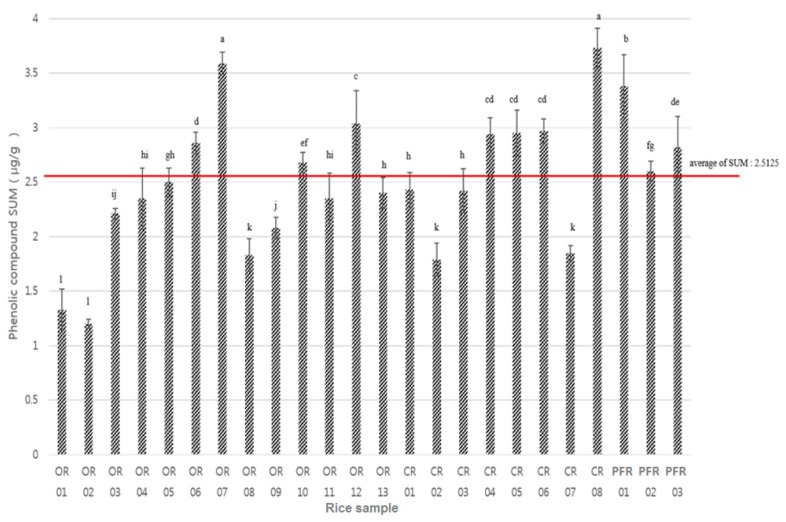
Distribution sum of the phenolic compounds in each sample. Letters a–l indicate a significant difference among the three groups (*p* < 0.0001).

Figure 5 shows a comparison of the quantities of the different phenolic compounds detected in the various samples of rice. Following the calculation of the average distribution sum of the phenolic compounds (i.e., 2.51 μg/g), it was apparent that a number of samples exceeded this average, namely, OR-06, -07, -10, and -12; CR-04, -05, -06, and -08; and PFR-01, -02, and -03. In addition, CR-08 showed the highest distribution sum of phenolic compounds, and was also the sample that contained the highest *p*-coumaric acid content.

**Figure 6:**
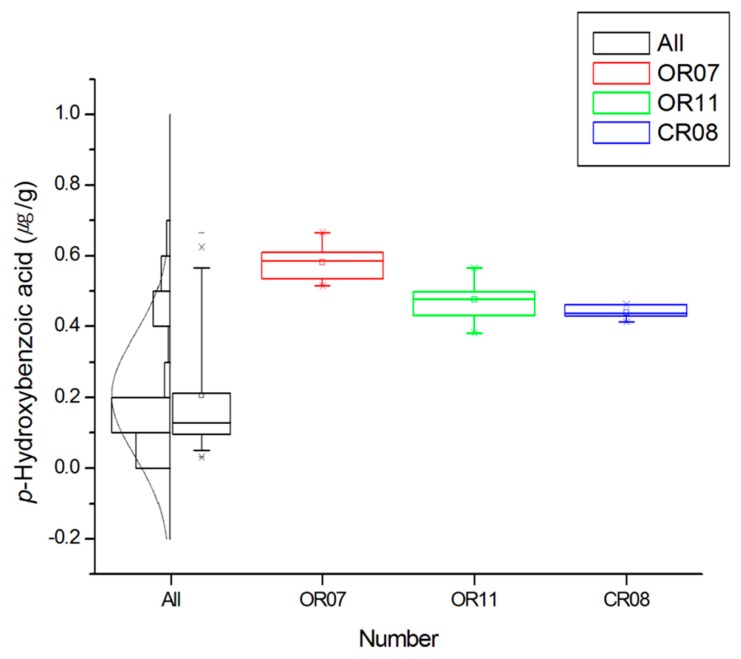
Boxplot, histogram, and distribution curve for the *p*-hydroxybenzoic acid content and for specific samples. Each box corresponds to the interquartile range containing the middle 50% of data. Whiskers indicate the highest and lowest values (95% and 5%, respectively) in the entire data range. The solid square within the box represents the mean value. The line across the box represents the median value.

In contrast, OR-02 contained the lowest quantity of phenolic compounds, in addition to the lowest salicylic, ferulic, protocatechuic, gentisic, and *p*-hydroxybenzoic acid contents. In addition, the average detected contents of eight phenolic compounds was found to decrease in the following order: ferulic > *p*-coumaric > caffeic > salicylic > *p*-hydroxybenzoic > gentisic > protocatechuic acids. In the case of protocatechuic and gentisic acids, low contents and low LOD values were common, while for *p*-hydroxybenzoic acid (Figure 6), higher values were apparent in a number of samples, namely, OR-07 (0.58 μg/g), OR-11 (0.48 μg/g), and CR-08 (0.44 μg/g).

It was also found that the salicylic acid contents were similar for all samples, with the exception of OR-07 (0.79 μg/g) and OR-11 (0.55 μg/g), which exhibited slightly higher values. In the case of caffeic acid, OR-07 (0.07 μg/g) and OR-11 (below the LOD) showed lower contents of the phenolic compounds, while PFR-01 (0.40 μg/g), PFR-02 (0.41 μg/g), PFR-03 (0.49 μg/g), and OR-06 (0.39 μg/g) gave higher values. In the case of *p*-coumaric acid, OR-01 (0.26 μg/g) exhibited a particularly low value, while OR-07 (1.02 μg/g) and CR-08 (1.11 μg/g) gave the highest contents. In addition, the highest and lowest ferulic acid contents were detected in PFR-01 (2.01 μg/g) and OR-02 (0.61 μg/g), respectively, while l-phenylalanine remained undetected in the majority of samples. Interestingly, PFR-01 (2.91 μg/g) exhibited the highest content of phenolic compounds. With the exception of the l-phenylalanine case, all other cases gave significant differences when *p* < 0.0001.

As the phenolic compounds tend to be concentrated in the bran layers, the loss of phenolic compounds can take place during separation of the seed coating and subsequent polishing. As such, brown rice was found to have a significantly higher ferulic acid content than white rice [9,31]. The abundance of secondary metabolites in rice and other crops is also affected by the environment (i.e., light, temperature, etc.) and by the genotype [32]. In a previous study, 14 varieties of rice were examined that contained a wide variety of phenolics, flavonoids, and anthocyanins [33]. Thus, to accurately examine the effect of the farming system, it is necessary to fix the varieties and to remove other influencing factors. However, in the case of our study, a mixture of varieties was employed, and thus genetic factors could not be removed. Moreover, as mentioned above, because greater secondary metabolite contents are found in brown rice, further experiments should be carried out using brown rice to more accurately examine the variation in secondary metabolite contents according to the farming system employed. Finally, a similar number of samples should be applied to increase the fairness of each group.

## 3. Materials and Methods

### 3.1. Rice Samples and Preparation 

Each polished OR (13 brands), PFR (3 brands), and CR (8 brands) sample was collected in triplicate (1–3 kg per replicate per brand) from eco-friendly farms and retail markets in Korea in 2016. All OR and PFR samples examined in this study were certified by pesticide residue screening test at certain inspection bodies designated by the National Agricultural Products Quality Management Service, Korea. All rice samples were lyophilized at <45 °C over four days (Freexeone 4.5, Labconco, Kansas City, MO, USA), and then pulverized to give the corresponding powders, which were stored in a desiccator at 23 °C and at a relative humidity of <20%.

### 3.2. Chemicals and Reagents

Among 56 phenolic compounds, isoflavonoids, phenylpropanoids, and stilbenoid standards were purchased from Sigma-Aldrich, Inc. (St. Louis, MO, USA), along with dimethyl sulfoxide (99.9%). The anthocyanin standards were purchased from Extrasynthese Impasse Jacquard, (Rue Jacquard, Genay, France). Biochanin A, daidzein, daidzin, formononetin, genistein, genistin, and glycitin were obtained from LC Laboratories (New Boston St. Woburn, MA, USA), while glycitein was purchased from Wako Pure Chemical Industries, Ltd. (Chuo-ku, Osaka, Japan), and polydatin (Piceid) and trans-resveratrol was obtained from Cayman Chemicals (Ann Arbor, MI, USA). HPLC grade methanol, absolute ethanol, and acetonitrile were purchased from Fisher Scientific (Seongnam-Si, Gyeonggi-Do, Korea), while formic acid (>98%) was obtained from Junsei Chemical Co., Ltd. (Chuo-ku, Tokyo, Japan). A 0.1 N standard solution of hydrochloric acid was purchased from Dae-Jung Chemicals & Metals Co., Ltd. (Shiheung-city, Gyeonggi-do, Korea), and deionized water with a resistivity of 18.2 MΩ was obtained from a PURELAB Option-Q System (Lane End, High Wycombe HP14 3BY, UK).

### 3.3. Extraction of Phenolic Compounds

Extraction of the phenolic compounds was carried out using a previously reported method [34,35]. More specifically, the desired powdered rice sample (1 g) was weighed in an Erlenmeyer flask, and acetonitrile (10 mL) and 0.1 N hydrochloric acid (2 mL) were added. The resulting mixture was allowed to stir at 23 °C for 2 h in a shaker (Green-SSeriker, Vision Scientific Co., Ltd., Gyeonggi-Do, Korea). After this time, the rice extract was filtered through Whatman filter paper (No. 42, 110 mm diameter) and the solvent evaporated at <33 °C under reduced pressure (EYELA SB-1200 rotary evaporator, Tokyo Rikakikai Co., Ltd., Tokyo, Japan). Subsequently, the obtained residue was re-dissolved in 80% aqueous methanol (5 mL), filtered through a 0.22 µm membrane filter (13 mm, PTFE, Thermo Scientific, Rochester, NY, USA), and subjected to liquid chromatography-electrospray ionization-tandem mass spectrometry (LC-ESI-MS/MS) for analysis of the phenolic compounds. The extraction was conducted in triplicate per each rice sample replicate.

### 3.4. Analysis of the Phenolic Compounds

The selected phenolic compounds present in rice samples of interest (OR, PFR, CR) were determined by LC-ESI-MS/MS system [36]. The HPLC system used in this study was from an Agilent HPLC System (Agilent Technologies, Santa Clara, CA, USA) equipped with a 1200 Series Degasser (G1322A), a 1260 Series Infinity Quaternary Pump VL (G1311C), a 1100 Series Autosampler (G1313A), and a 1100 Series Column Compartment (G1316A). A reversed-phase analytical column (Thermo Scientific™ Syncronis^TM^ C18, 4.6 × 150 mm, 5 μm) was used for phenolic compound separation. Mobile phases A and B were composed of 0.1% formic acid in deionized water and 0.1% formic acid in acetonitrile, respectively. The gradient program employed was as follows: 0–10 min, A 90% B 10%; 10–20 min, A 60% B 40%; 20–25 min, A 50% B 50%; 25–26 min, A 0% B 100%; 26–30 min, A 90% B 10% (for re-equilibrium). The flow rate was set at 0.5 mL/min and the column was maintained at 23 °C. An aliquot of each sample solution (10 μL) was injected into the system. 

The mass spectra were obtained using an API 2000 System (AB Sciex, Framingham, MA, USA) equipped with an ESI source and a triple-quadrupole mass spectrometer (MS/MS). The ESI source and the MS/MS were operated in the negative ion and multiple reaction monitoring (MRM) modes, respectively. The optimum MRM parameter for each targeted compound was acquired using the infusion mode with the corresponding purchased standard solution (Table S1, see online supplementary material). The optimum ESI parameters determined by flow injection analysis (FIA) were as follows: Curtain gas (CUR) 50 psi, collision gas (CAD) 2 psi, ion-spray voltage (IS) −4400 V, ion-spray probe temperature (TEM) 500 °C ion source gas 1 (GS1) 40 psi, ion source gas 2 (GS2) 50 psi, and interface heater turned on. Nitrogen was used as the curtain, collision, nebulizer, and heater gas. The ion-spray probe was aligned from the default position at 3 mm and 5 mm along the vertical and horizontal axes, respectively. Total LC-MS/MS analytical time was 30 min per sample, and the analysis was carried out in triplicate per each sample replicate.

Identification of the analytes was accomplished in MRM mode by comparing the retention times (RT) and *m/z* (mass-to-charge ratio) values of the molecular and product ions (Q1 and Q3 values in Table S1, see online supplementary material) with the corresponding standard solutions. For preparation of the stock solutions, all standards were dissolved using the appropriate solvents according to their physical/chemical properties. The obtained standard solutions were diluted in series using 80% methanol in deionized water to construct their corresponding calibration curves. The MRM ion chromatograms of the 56 selected phenolic compounds and free amino acids are shown in Figure S2 (see online supplementary material). In addition, the mass spectra of the representative phenolic metabolites along with their fragmentation patterns, are given in Figure S3 (see online supplementary material). 

A quantitative analysis of the data and the process employed for establishing the external calibration curve (Table S2, see online supplementary material) was carried out using Analyst software (version 1.6.2; AB Sciex, Framingham, MA, USA). In this study, all calibration curves exhibited good linearity (r^2^ = 0.99) in the range of 0.01–5 µg·mL^−1^. Further, for the purpose of evaluation of external calibration curve fitness, the residual that indicates the difference between measured and calculated peak areas also showed less than ±8% in the concentration ranges of all external calibration curves created in this study. Besides, the repeatability and reproducibility based on the analyses of phenolic standards and sample extracts also exhibited ~5% (*n* = 3) in this study. The LOD and LOQ values were calculated with signal-to-noise (S/N) ratios of 3 and 10 (Table S2, see online supplementary material), and all values are expressed as μg/g on a dry weight basis. Meanwhile, the matrix effect during our LC-MS/MS analysis was observed in less than ±5% (Figure S4, see online supplementary material).

### 3.5. Statistical Analysis

Statistical analysis of the combined replicate data was performed using Statistical Analysis System (SAS) software (version 9.4; SAS Institute Inc., NC, USA). The ANOVA (analysis of variance) and the least significant difference (LSD) tests were conducted at the 0.05 probability level. 

## 4. Conclusions

As the different quantities of secondary metabolites, such as phenolic compounds, are predicted to play a major role in the defense systems of plants, we herein wished to investigate variations in the phenolic compounds present in organic rice (OR), conventional rice (CR), and pesticide-free rice (PFR), as the contents of such compounds are known to vary upon the use of organic synthetic pesticides and chemical fertilizers. In this case, seven phenolic compounds were detected in addition to l-phenylalanine, a precursor of such phenolics. When classified according to the farming system employed, ferulic acid was found to be the most abundant compound, decreasing in the following order: PFR > CR > OR (*p* < 0.0001). When classified according to the brand (or sample number), CR-08 and OR-02 exhibited the highest and lowest distribution sums of the various phenolic compounds, respectively. In addition, when classified once again according to the farming system, the highest phenolic content was found for PFR, followed by CR and OR (*p* < 0.0001). Interestingly, the obtained results indicated that the contents of the individual phenolic compounds in polished rice samples were particularly low, but were significant when analyzed based on the sum of components present. However, further studies are required to improve the fairness of these experiments.

## Figures and Tables

**Table 1:** Phenolic components present in the rice samples obtained using different farming systems (μg/g on a dry weight basis, injection and extraction *n* = 3).

Sample Name	PCA	GTA	*p*HBA	SA	LPA	*p*CMA	CA	FA	Distribution Sum of Phenolic Compound
OR 01	<LOD	<LOD	<LOD	0.24 ± 0.03	ND	0.26 ± 0.08	0.14 ± 0.01	0.69 ± 0.10	1.33 ± 0.19
OR 02	<LOD	<LOD	<LOD	0.13 ± 0.01	ND	0.27 ± 0.04	0.19 ± 0.01	0.61 ± 0.02	1.20 ± 0.04
OR 03	0.02 ± 0.02	0.07 ± 0.00	0.09 ± 0.02	0.15 ± 0.01	<LOD	0.53 ± 0.03	0.18 ± 0.00	1.17 ± 0.04	2.21 ± 0.05
OR 04	0.03 ± 0.01	0.09 ± 0.01	0.10 ± 0.02	0.22 ± 0.04	2.59 ± 0.22	0.42 ± 0.05	0.25 ± 0.01	1.26 ± 0.16	2.35 ± 0.28
OR 05	0.03 ± 0.03	0.03 ± 0.00	0.13 ± 0.02	0.18 ± 0.01	<LOD	0.51 ± 0.07	0.26 ± 0.01	1.36 ± 0.08	2.50 ± 0.13
OR 06	0.02 ± 0.01	0.03 ± 0.00	<LOD	0.24 ± 0.01	<LOD	0.43 ± 0.04	0.39 ± 0.01	1.76 ± 0.09	2.86 ± 0.10
OR 07	0.03 ± 0.01	<LOD	0.58 ± 0.05	0.79 ± 0.03	<LOD	1.02 ± 0.04	0.07 ± 0.01	1.10 ± 0.08	3.59 ± 0.10
OR 08	<LOD	<LOD	<LOD	0.22 ± 0.02	ND	0.31 ± 0.03	0.13 ± 0.01	1.16 ± 0.13	1.83 ± 0.15
OR 09	<LOD	0.04 ± 0.00	0.18 ± 0.03	0.23 ± 0.01	<LOD	0.40 ± 0.03	0.12 ± 0.01	1.11 ± 0.09	2.08 ± 0.10
OR 10	0.05 ± 0.01	0.04 ± 0.01	0.09 ± 0.01	0.23 ± 0.01	ND	0.47 ± 0.03	0.34 ± 0.02	1.46 ± 0.10	2.68 ± 0.09
OR 11	<LOD	<LOD	0.48 ± 0.06	0.55 ± 0.04	ND	0.47 ± 0.05	<LOD	0.85 ± 0.10	2.35 ± 0.23
OR 12	0.06 ± 0.02	<LOD	<LOD	0.32 ± 0.04	ND	0.52 ± 0.07	0.36 ± 0.03	1.78 ± 0.21	3.04 ± 0.30
OR 13	<LOD	<LOD	<LOD	0.24 ± 0.02	ND	0.42 ± 0.04	0.32 ± 0.02	1.42 ± 0.11	2.40 ± 0.14
Group Mean	0.03 ± 0.02	0.05 ± 0.02 ^b^	0.23 ± 0.19 ^a^	0.29 ± 0.18 ^a^	2.59 ± 0.22 ^b^	0.47 ± 0.19 ^ab^	0.23 ± 0.1 ^c^	1.21 ± 0.36 ^c^	2.34 ± 0.65 ^c^
CR 01	<LOD	<LOD	<LOD	0.23 ± 0.01	ND	0.40 ± 0.05	0.35 ± 0.01	1.44 ± 0.11	2.43 ± 0.16
CR 02	<LOD	0.05 ± 0.01	<LOD	0.12 ± 0.00	<LOD	0.29 ± 0.06	0.23 ± 0.02	1.11 ± 0.11	1.79 ± 0.15
CR 03	<LOD	<LOD	<LOD	0.21 ± 0.01	ND	0.36 ± 0.03	0.33 ± 0.01	1.52 ± 0.18	2.42 ± 0.20
CR 04	0.03 ± 0.02	0.06 ± 0.01	0.19 ± 0.03	0.21 ± 0.01	2.74 ± 0.09	0.56 ± 0.08	0.30 ± 0.01	1.59 ± 0.12	2.94 ± 0.15
CR 05	<LOD	0.05 ± 0.01	0.14 ± 0.01	0.16 ± 0.01	<LOD	0.68 ± 0.10	0.27 ± 0.01	1.66 ± 0.13	2.95 ± 0.21
CR 06	<LOD	<LOD	0.12 ± 0.02	0.28 ± 0.01	ND	0.55 ± 0.03	0.31 ± 0.01	1.71 ± 0.11	2.97 ± 0.11
CR 07	<LOD	<LOD	<LOD	0.15 ± 0.00	<LOD	0.40 ± 0.03	0.24 ± 0.02	1.07 ± 0.07	1.85 ± 0.07
CR 08	0.05 ± 0.01	0.06 ± 0.00	0.44 ± 0.02	0.37 ± 0.04	<LOD	1.11 ± 0.09	0.22 ± 0.01	1.47 ± 0.10	3.73 ± 0.18
Group Mean	0.04 ± 0.02	0.05 ± 0.01 ^b^	0.22 ± 0.13 ^a^	0.22 ± 0.08 ^b^	2.74 ± 0.09 ^ab^	0.54 ± 0.26 ^a^	0.28 ± 0.05 ^b^	1.45 ± 0.25 ^b^	2.63 ± 0.63 ^b^
PFR 01	<LOD	0.14 ± 0.01	0.06 ± 0.02	0.25 ± 0.01	2.91 ± 0.14	0.51 ± 0.05	0.40 ± 0.04	2.01 ± 0.19	3.38 ± 0.29
PFR 02	0.04 ± 0.01	0.04 ± 0.01	<LOD	0.14 ± 0.01	2.87 ± 0.08	0.48 ± 0.03	0.41 ± 0.02	1.49 ± 0.09	2.6 ± 0.09
PFR 03	0.02 ± 0.01	0.08 ± 0.01	0.09 ± 0.04	0.21 ± 0.02	2.67 ± 0.25	0.37 ± 0.06	0.49 ± 0.09	1.56 ± 0.30	2.82 ± 0.28
Group Mean	0.03 ± 0.01	0.08 ± 0.04 ^a^	0.08 ± 0.03 ^b^	0.2 ± 0.05 ^b^	2.82 ± 0.2 ^a^	0.45 ± 0.08 ^b^	0.44 ± 0.07 ^a^	1.69 ± 0.31 ^a^	2.93 ± 0.41 ^a^
LSD	0.0113	0.0125	0.0822	0.0551	0.1575	0.08	0.033	0.1263	0.2423

Data are expressed as mean ± standard deviation. Different superscripts (a–c) mean a significant difference among three groups (*p* < 0.05). Abbreviations: PCA (protocatechuic acid), GTA (gentisic acid), pHBA (*p*-hydroxybenzoic acid), pCMA (*p*-coumaric acid), FA (ferulic acid), SA (salicylic acid), CA (caffeic acid), LPA (l-phenylalanine), <LOD (less than the limit of detection), ND (not detected), organic rice (OR), pesticide-free rice (PFR), and conventional rice (CR).

## Supplementary Materials

The following are available online at http://www.mdpi.com/1420-3049/24/1/67/s1. Figures representing the steps of shikimic acid pathway; MRM ion chromatogram of the 56 selected phenolic compounds and free amino acids; MS/MS spectra of the selected phenolic standard and free amino acids; matrix effect evaluation during LC-MS/MS analysis; tables outlining the MRM parameters for the 56 selected phenolic compounds and free amino acids; and calibration curves of the eight detected phenolic compounds and free amino acids in the rice grain.
