# Supplementary material for: Analysis of Selected Phenolic Compounds in Organic, Pesticide-Free, Conventional Rice (Oryza sativa L.) Using LC-ESI-MS/MS
Source: Molecules. 2018 Dec 25;24(1):67. doi: 10.3390/molecules24010067 (PMC6337394; doi:10.3390/molecules24010067)
Supplement: Supplementary file 1 [file molecules-24-00067-s001.pdf]

Electronic Supplementary Information (ESI) for Molecules

Analysis of selected phenolic compounds in organic, pesticide-free, conventional rice (*Oryza sativa* L.) using LC-ESI-MS/MS

Mayakrishnan Prabakaran[1], Ill-Min Chung[1], Na-Young Son[1], Hee-Youn Chi[1], So-Yeon Kim[1], Yu-Jin Yang[1], Chang Kwon[1], Yeon-Ju An[1], Ateeque Ahmad[2], Seung-Hyun Kim[1,*]

[1]Department of Crop Science, College of Sanghuh Life Science, Konkuk University, Seoul 05029, Republic of Korea; prabakarannitt@gmail.com (M.P.); imcim@konkuk.ac.kr (I.-M.C.); sonkittyy@konkuk.ac.kr (N.-Y.S); chi1143@konkuk.ac.kr (H.-Y.C); hellosy1@konkuk.ac.kr (S.-Y.K.); jin9031@konkuk.ac.kr (Y.-J.Y.); chang794@konkuk.ac.kr (C.K.); ayj3043@konkuk.ac.kr (Y.-J.A.); kshkim@konkuk.ac.kr (S-H.K.)

[2]Process Chemistry and Technology Department, CSIR-Central Institute of Medicinal and Aromatic Plants, Lucknow 226015, India; ateeque97@gmail.com (A.A.)

*Correspondence: kshkim@konkuk.ac.kr; Tel.: +82-02-2049-6163; Fax: +82-02-455-1044

Table S1. Optimized MRM parameters for 56 selected phenolic compounds and free amino acid.

Type	Compound	Formula	Q1 [M-H]⁻ , *m/z*	Q3, *m/z*	DP (volt)	FP (volt)	EP (volt)	CEP (volt)	CE (volt)	CXP (volt)
Phenolic acid	Gallic acid	$C_7H_6O_5$	169.1	125.0	-26.0	-350.0	-10.0	-8.0	-20.0	-6.0
	Protocatechuic acid	$C_7H_6O_4$	152.9	108.9	-16.0	-330.0	-9.0	-10.0	-22.0	-6.0
	Gentisic acid	$C_7H_6O_4$	152.9	107.9	-16.0	-350.0	-8.5	-6.0	-28.0	-22.0
	p-Hydroxybenzoic acid	$C_7H_6O_3$	136.9	92.9	-16.0	-350.0	-8.5	-12.0	-24.0	-6.0
	Syringic acid	$C_9H_{10}O_5$	196.9	182.0	-16.0	-350.0	-10.5	-12.0	-30.0	-6.0
	Vanillin	$C_8H_8O_3$	150.9	135.9	-16.0	-280.0	-4.0	-10.0	-18.0	-28.0
	3,4-Dimethoxybenzoic acid	$C_9H_{10}O_4$	181.0	136.9	-16.0	-350.0	-7.5	-12.0	-18.0	-8.0
	2,4-Dihydroxybenzoic acid	$C_7H_6O_4$	152.9	108.9	-21.0	-350.0	-4.5	-10.0	-18.0	-6.0
	5-Sulfosalicylic acid	$C_7H_6O_6S$	216.8	198.8	-21.0	-320.0	-4.5	-10.0	-22.0	-8.0
	Homogentisic acid	$C_8H_8O_4$	166.9	122.9	-21.0	-300.0	-7.0	-14.0	-14.0	-28.0
	Salicylic acid	$C_7H_6O_3$	136.9	92.9	-16.0	-320.0	-7.5	-12.0	-22.0	-6.0
	Vanillic acid	$C_8H_8O_4$	166.9	151.9	-11.0	-250.0	-6.5	-10.0	-18.0	-32.0
Flavonoid	Catechin	$C_{15}H_{14}O_6$	288.8	108.9	-21.0	-340.0	-9.0	-16.0	-34.0	-4.0
	Rutin	$C_{27}H_{30}O_{16}$	609.0	299.7	-91.0	-350.0	-10.5	-34.0	-52.0	-14.0
	Naringin	$C_{27}H_{32}O_{14}$	579.0	151.1	-126.0	-310.0	-10.5	-28.0	-52.0	-32.0
	Myricetin	$C_{15}H_{10}O_8$	316.8	151.1	-61.0	-310.0	-10.0	-16.0	-36.0	-28.0
	Quercetin	$C_{15}H_{10}O_7$	300.9	150.8	-31.0	-330.0	-10.5	-18.0	-28.0	-32.0
	Naringenin	$C_{15}H_{12}O_5$	270.8	64.7	-66.0	-90.0	-8.0	-16.0	-30.0	-26.0
	Kaempferol	$C_{15}H_{10}O_6$	284.8	65.0	-81.0	-270.0	-8.5	-20.0	-72.0	-12.0
	Hesperetin	$C_{16}H_{14}O_6$	301.0	163.7	-51.0	-310.0	-7.5	-20.0	-32.0	-32.0
	Orientin	$C_{21}H_{20}O_{11}$	447.1	327.0	-61.0	-220.0	-11.0	-20.0	-22.0	-54.0
	Vitexin	$C_{21}H_{20}O_{10}$	430.8	310.8	-56.0	-260.0	-9.0	-28.0	-22.0	-50.0
	Apigenin	$C_{15}H_{10}O_5$	268.9	117.0	-46.0	-350.0	-10.0	-20.0	-56.0	-8.0
	Luteolin	$C_{15}H_{10}O_6$	285.0	133.2	-66.0	-250.0	-10.0	-16.0	-46.0	-30.0
Anthocyanin	Cyanidin chloride	$C_{15}H_{11}ClO_6$	320.9	284.9	-1.0	-290.0	-4.5	-16.0	-10.0	-54.0
	Delphinidin chloride	$C_{15}H_{11}ClO_7$	336.9	300.8	-1.0	-290.0	-4.5	-14.0	-10.0	-56.0
	Malvidin chloride	$C_{17}H_{15}ClO_7$	365.0	328.9	-6.0	-350.0	-3.5	-14.0	-14.0	-10.0
	Pelargonidin chloride	$C_{15}H_{11}ClO_5$	305.0	268.9	-1.0	-270.0	-4.0	-14.0	-12.0	-52.0
	Peonidin chloride	$C_{16}H_{13}ClO_6$	334.9	298.8	-1.0	-330.0	-3.0	-14.0	-12.0	-56.0
	Peonidin 3-O-*β* glucoside chloride	$C_{22}H_{23}ClO_{11}$	497.0	298.9	-1.0	-350.0	-8.0	-26.0	-32.0	-54.0

Table S1. *Continued.*

Type	Compound	Formula	Q1 [M-H]⁻ , *m/z*	Q3, *m/z*	DP (volt)	FP (volt)	EP (volt)	CEP (volt)	CE (volt)	CXP (volt)
Stilbenoid	*trans*-Resveratrol	$C_{14}H_{12}O_3$	226.9	142.8	-56.0	-170.0	-11.0	-14.0	-34.0	-32.0
	cis-Resveratrol	$C_{14}H_{12}O_3$	226.9	142.8	-61.0	-330.0	-10.5	-14.2	-34.0	-28.0
	Polydatin	$C_{20}H_{22}O_8$	389.0	226.8	-71.0	-320.0	-10.5	-18.2	-20.0	-48.0
Isoflavonoid	Formononetin	$C_{16}H_{12}O_4$	266.8	251.8	-21.0	-290.0	-7.5	-14.0	-24.0	-44.0
	Biochanin A	$C_{16}H_{12}O_5$	282.8	267.9	-46.0	-210.0	-8.0	-14.0	-24.0	-48.0
	Genistein	$C_{15}H_{10}O_5$	268.9	132.9	-76.0	-240.0	-10.5	-12.0	-44.0	-6.0
	Genistin	$C_{21}H_{20}O_{10}$	431.1	268.0	-81.0	-140.0	-9.5	-64.0	-44.0	-12.0
	Glycitin	$C_{22}H_{22}O_{10}$	445.1	238.7	-81.0	-300.0	-8.0	-22.0	-42.0	-52.0
	Glycitein	$C_{16}H_{12}O_5$	282.9	267.8	-31.0	-340.0	-7.0	-54.0	-26.0	-12.0
	Daidzin	$C_{21}H_{20}O_9$	415.0	251.9	-66.0	-320.0	-10.0	-28.0	-38.0	-46.0
	Daidzein	$C_{15}H_{10}O_4$	252.9	131.9	-76.0	-350.0	-10.5	-26.0	-54.0	-6.0
	Acetyl Daidzin	$C_{23}H_{22}O_{10}$	456.9	252.0	-86.0	-350.0	-10.0	-54.0	-44.0	-12.0
	Acetyl Genistin	$C_{23}H_{22}O_{11}$	473.0	267.9	-101.0	-230.0	-10.5	-30.0	-40.0	-8.0
	Acetyl Glycitin	$C_{24}H_{24}O_{11}$	487.0	281.9	-76.0	-350.0	-10.5	-30.0	-36.0	-10.0
	Malonyl Daidzin	$C_{24}H_{22}O_{12}$	501.0	252.7	-6.0	-330.0	-4.0	-21.0	-22.0	-50.0
	Malonyl Genistin	$C_{24}H_{22}O_{13}$	517.0	268.7	-11.0	-340.0	-4.5	-22.0	-20.0	-8.0
	Malonyl Glycitin	$C_{25}H_{24}O_{13}$	531.0	282.9	-26.0	-340.0	-5.5	-21.8	-22.0	-48.0
Phenylpropanoid	Chlorogenic acid	$C_{16}H_{18}O_9$	352.8	191.0	-21.0	-330.0	-7.0	-32.0	-30.0	-10.0
	p-Coumaric acid	$C_9H_8O_3$	162.8	118.7	-11.0	-340.0	-7.0	-14.0	-20.0	-6.0
	Ferulic acid	$C_{10}H_{10}O_4$	192.9	133.9	-11.0	-350.0	-10.0	-12.0	-22.0	-8.0
	m-Coumaric acid	$C_9H_8O_3$	162.8	119.0	-21.0	-330.0	-9.0	-8.0	-22.0	-4.0
	o-Coumaric acid	$C_9H_8O_3$	162.8	118.9	-6.0	-290.0	-11.5	-14.0	-18.0	-4.0
	trans-Cinnamic acid	$C_9H_8O_2$	146.9	102.9	-26.0	-310.0	-7.0	-10.0	-14.0	-20.0
	Caffeic acid	$C_9H_8O_4$	178.9	134.8	-16.0	-330.0	-8.0	-14.0	-22.0	-6.0
Amino acid	L-Phenylalanine	$C_9H_{11}NO_2$	163.9	146.8	-41.0	-310.0	-10.5	-12.0	-18.0	-10.0
	L-Tyrosine	$C_9H_{11}NO_3$	179.9	163.0	-71.0	-250.0	-10.0	-20.0	-14.0	-8.0

Abbreviations are as follows: Q1 (molecular ion mass), Q3 (product ion mass for quanrification), DP (declustering potential), FP (focusing potential), EP (entrance potential), CEP (collision cell entrance potential), CE (collision energy), and CXP (collision cell exit potential).

Table S2. Calibration curves of 8 detected phenolic compounds and free amino acid in rice grain.

Type	Compound	RT	Concentration range (μg/mL)	Regression Equation	R^2	LOD (μg/g)	LOQ (μg/g)
Phenolic acid	Protocatechuic acid	9.15	0.01 - 1	y= 1430000x + 4580	1.00	0.004	0.013
	Gentisic acid	11.77	0.01 - 1	y= 1070000x + 967	1.00	0.005	0.017
	p-Hydroxybenzoic acid	11.27	0.05 - 1	y= 715000x + 21700	1.00	0.011	0.037
	Salicylic acid	18.04	0.01 - 0.5	y= 4300000x + 14500	1.00	0.005	0.017
Phenylpropanoid	*p*-Coumaric acid	13.52	0.05 - 1	y= 1200000x 53600	1.00	0.005	0.017
	Caffeic acid	11.43	0.01 - 0.1	y= 2290000x + 4410	1.00	0.008	0.027
	Ferulic acid	13.95	0.1 - 1	y= 168000x + 3870	0.99	0.033	0.110
Amino acid	L-Phenylalanine	6.30	0.5 - 5	$y= -7050x^2 + 53700x -15500$	0.99	0.5	1.667

Abbreviations are as follows: RT (retention time), R^2 (coefficient of determination), LOD (the limit of detection), and LOQ (the limit of quantization).

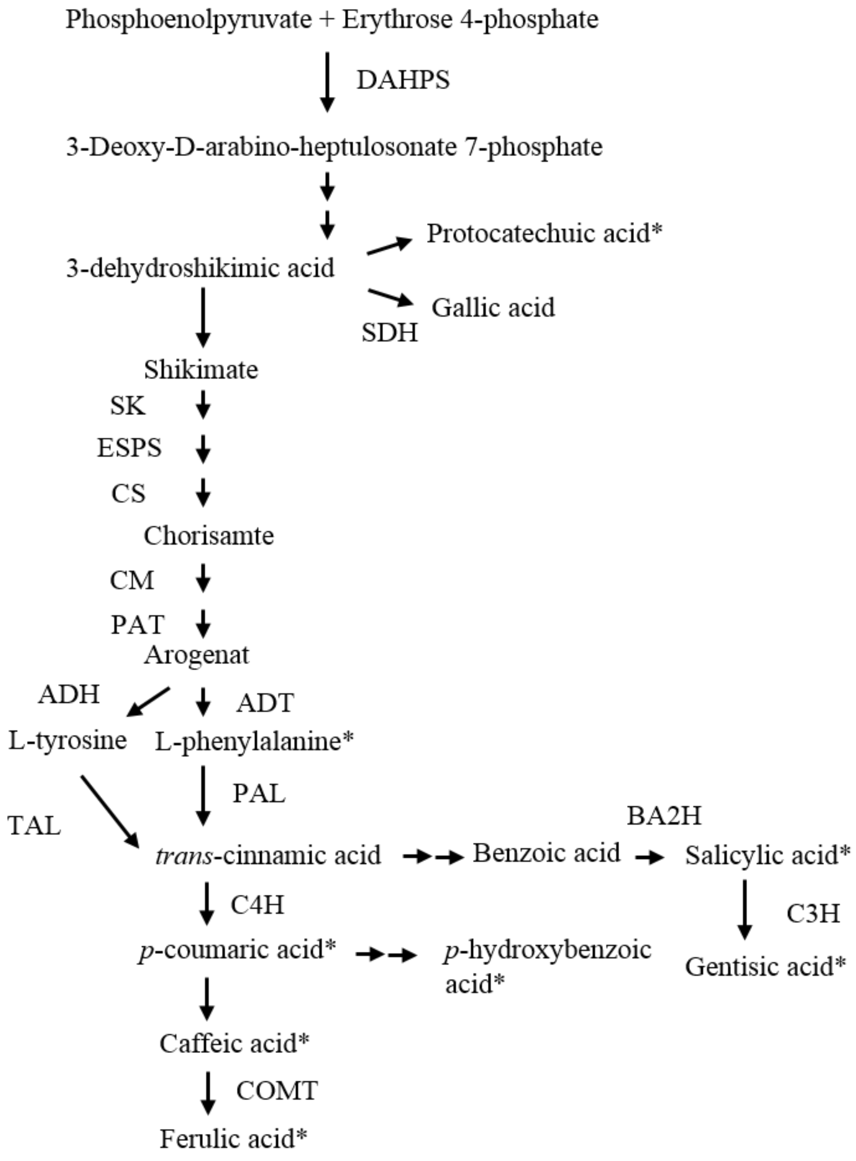

Figure S1. Phenolic acid synthesis by shikimic acid pathway. Abbreviations: DAHPS (3-deoxy-D-arabino-heptulosonate 7-phosphate synthase), SDH (shikimate dehydrogenases), SK (shikimate kinase), ESPS (5-enolpyruvylshikimate 3-phosphatesynthase), CS (chorismate synthase), CM (chorismate mutase), PAT (prephenate aminotransferase), ADH (arogenate dehydrogenase), ADT (arogenate dehydratase), PAL (phenylalanine ammonia-lyase), C4H (cinnamte 4-hydroxylase), BA2H (benzoic acid 2-hydroxylase), S3H (salicylic acid 3-hydroxylase), COMT (caffeic acid/5-hydroxy ferulic acid-O-methyltransferase).

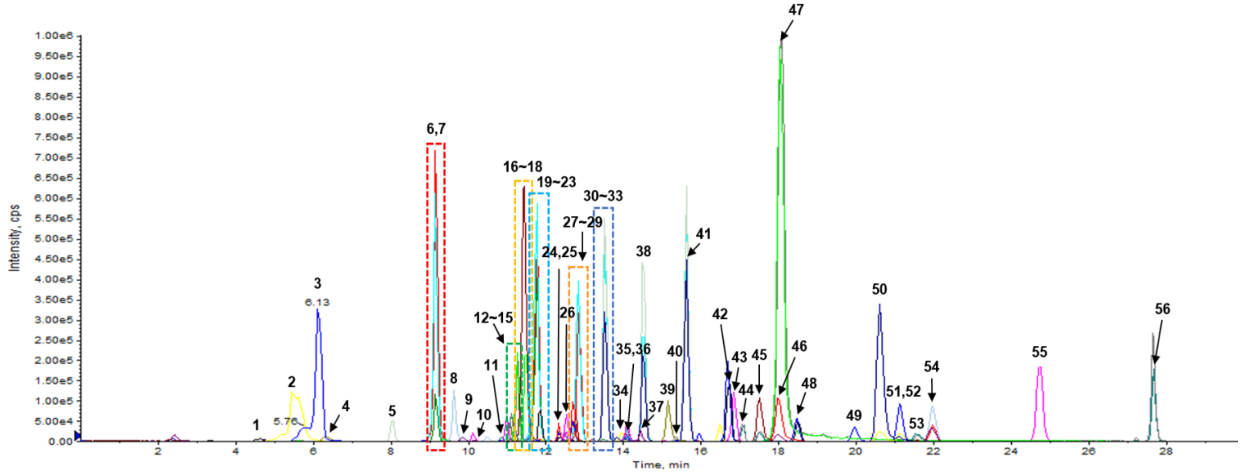

Figure S2. MRM ion chromatogram of 56 selected phenolic compounds and free amino acids standards. 1. L-Tyrosine; 2. 5-Sulfosalicylic acid; 3. Gallic acid; 4. L-Phenylalanine; 5. Homogentisic acid; 6. Protocatechuic acid; 7. Peonidin 3-O-*β* glucoside chloride; 8. Chlorogenic acid; 9. Delphinidin chloride; 10. Catechin; 11. Cyanidin chloride; 12. Daidzin; 13. Glycitin; 14. Orientin; 15. *p*-Hydroxybenzoic acid; 16. Caffeic acid; 17. Rutin; 18. Syringic acid; 19. Gentisic acid; 20. Vitexin; 21. Pelargonidin chloride; 22. Malvidin chloride; 23. Peonidin chloride; 24. Polydatin (Piceid); 25. Malonyl Glycitin; 26. Malonyl Daidzin; 27. Genistin; 28. Naringin; 29. *β*-Resorcylic acid; 30. Acetyl Daidzin; 31. *p*-Coumaric acid; 32. Acetyl Glycitin; 33. Vanillic acid; 34. Ferulic acid; 35. Malonyl Genistin; 36. Vanillin; 37. Veratric acid; 38. *m*-Coumaric acid; 39. Myricetin; 40. Acetyl Genistin; 41. *o*-Coumaric acid; 42. *trans*-Resveratrol; 43. Daidzein; 44. Glycitein; 45. Luteolin; 46. Quercetin; 47. Salicylic acid; 48. *cis*-Resveratrol; 49. *trans*-Cinnamic acid; 50. Apigenin; 51. Naringenin; 52. Genistein; 53. Kaempferol; 54. Hesperetin; 55. Formononetin; 56. Biochanin A.

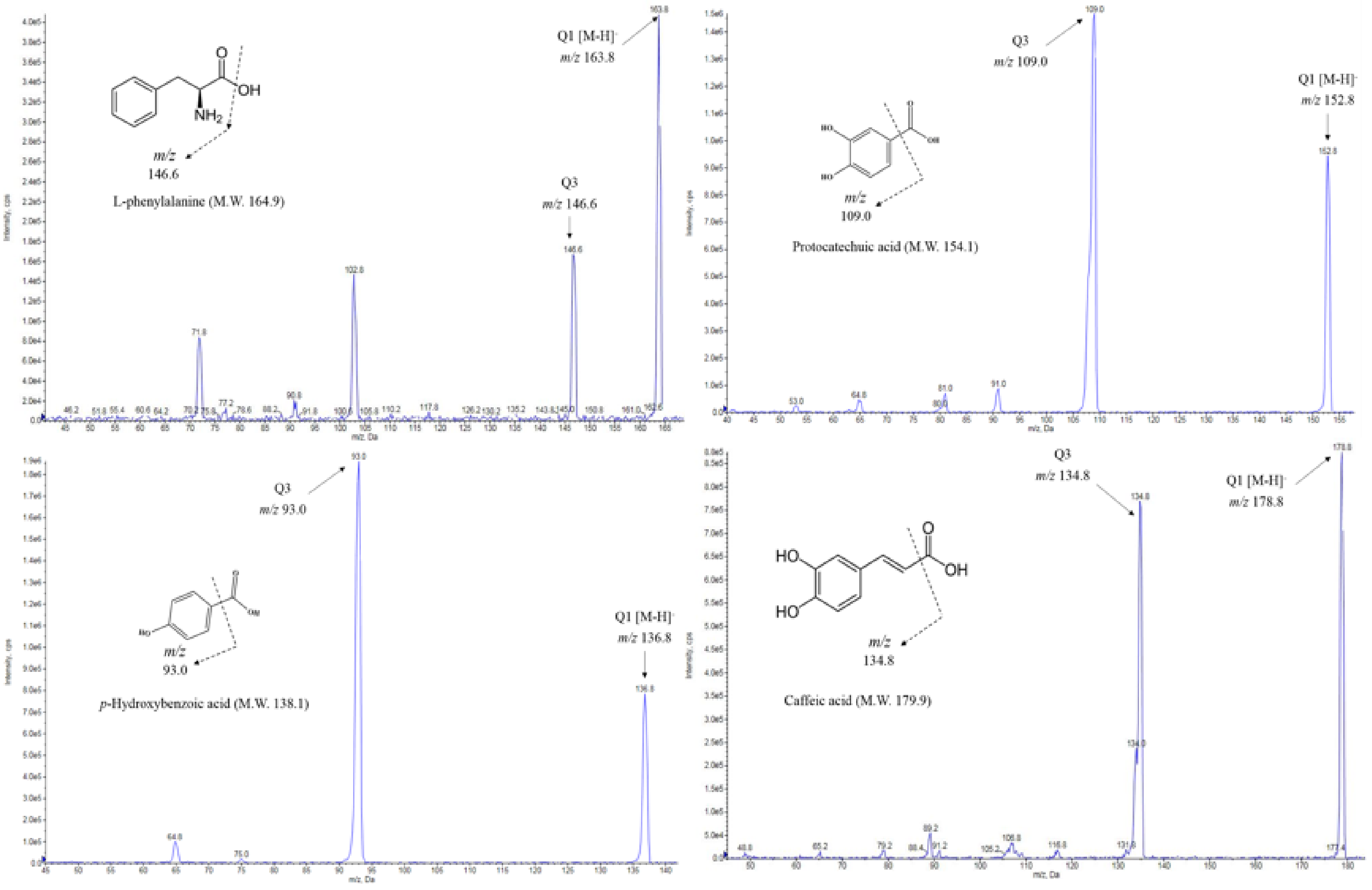

Q1 [M-H]-
m/z 163.8
m/z
146.6
L-phenylalanine (M.W. 164.9)
Q3
m/z 146.6
Q3
m/z 109.0
Q1 [M-H]-
m/z 152.8
m/z
109.0
Protocatechuic acid (M.W. 154.1)
Q3
m/z 93.0
Q1 [M-H]-
m/z 136.8
m/z
93.0
p-Hydroxybenzoic acid (M.W. 138.1)
Q3
m/z 134.8
Q1 [M-H]-
m/z 178.8
m/z
134.8
Caffeic acid (M.W. 179.9)

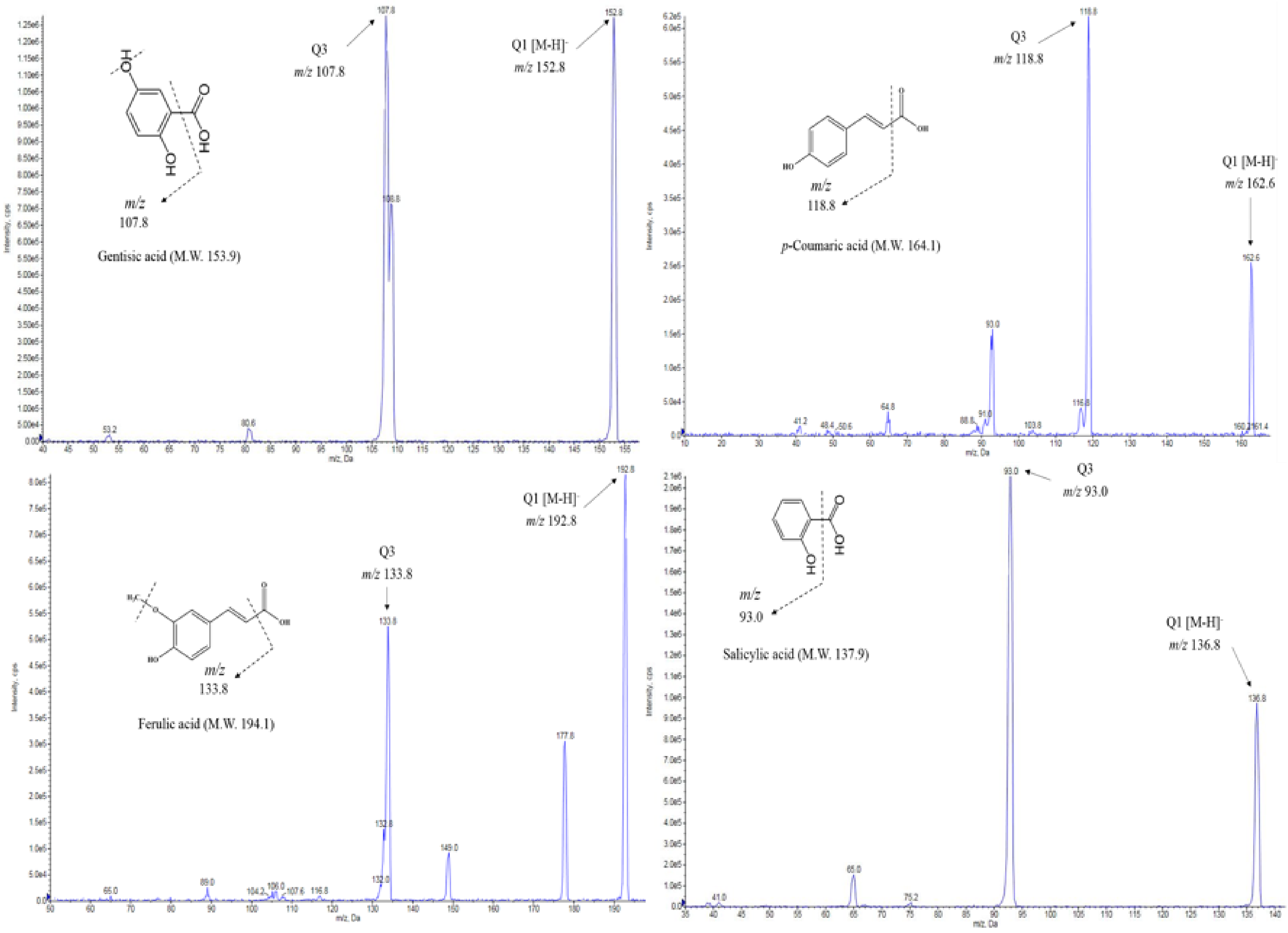

Figure S3. Representative MS/MS spectra of the phenolic standard and free amino acid.

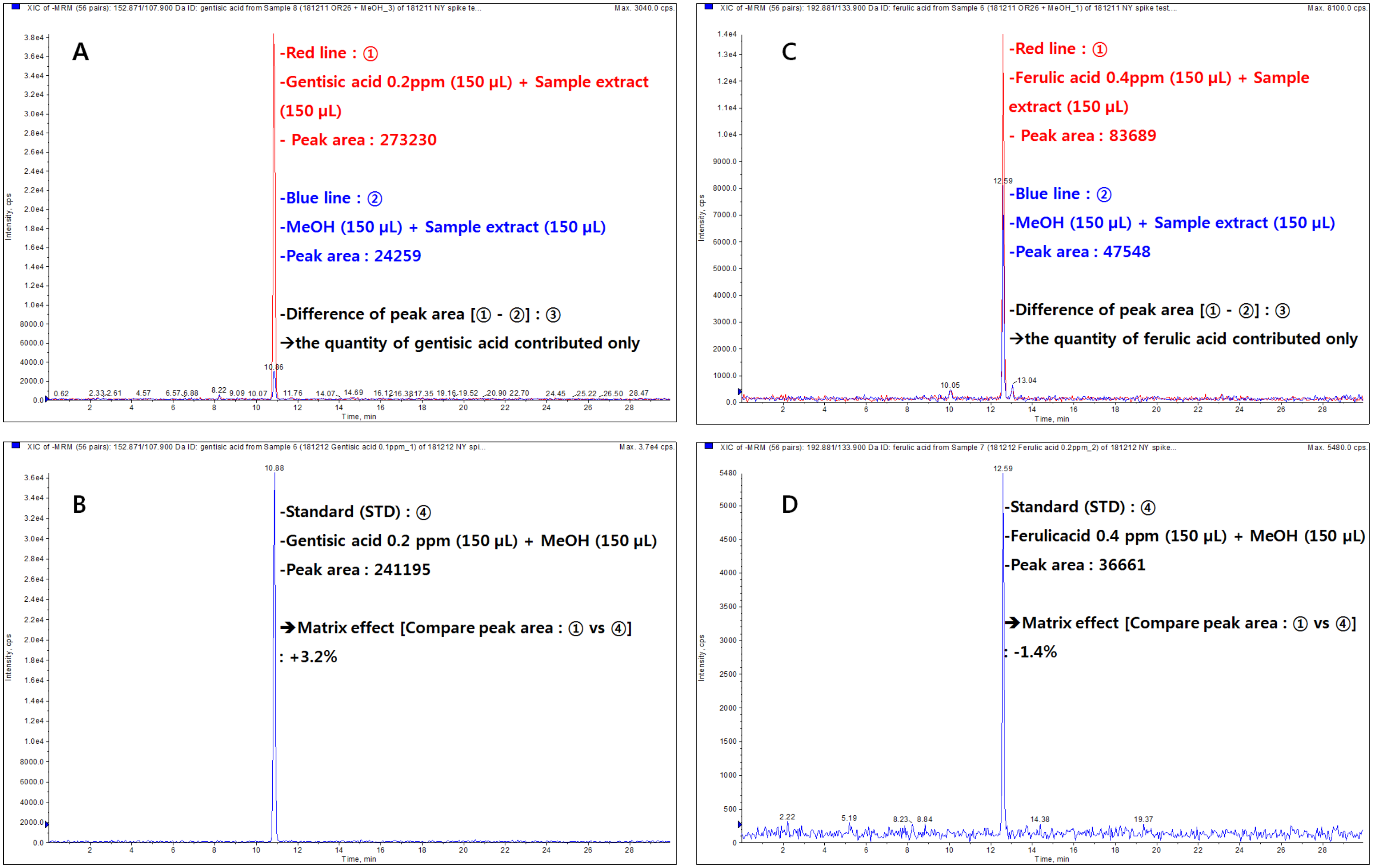

Figure S4. Representative ion chromatograms of matrix effect for response (peak area) of genitisic (0.1 μg·mL^{-1}) and ferulic acids (0.2 μg·mL^{-1}) by using LC-MS/MS.
